# Tolerance of *Ruppia sinensis* Seeds to Desiccation, Low Temperature, and High Salinity With Special Reference to Long-Term Seed Storage

**DOI:** 10.3389/fpls.2018.00221

**Published:** 2018-03-23

**Authors:** Ruiting Gu, Yi Zhou, Xiaoyue Song, Shaochun Xu, Xiaomei Zhang, Haiying Lin, Shuai Xu, Shidong Yue, Shuyu Zhu

**Affiliations:** ^1^CAS Key Laboratory of Marine Ecology and Environmental Sciences, Institute of Oceanology, Chinese Academy of Sciences, Qingdao, China; ^2^Laboratory for Marine Ecology and Environmental Science, Qingdao National Laboratory for Marine Science and Technology, Qingdao, China; ^3^College of Earth Sciences, University of Chinese Academy of Sciences, Beijing, China; ^4^State Key Laboratory of Water Environment Simulation, School of Environment, Beijing Normal University, Beijing, China; ^5^Yellow River Delta National Nature Reserve Management Bureau, Dongying, China

**Keywords:** desiccation, morphology, seagrass, *Ruppia sinensis*, seed, storage, salinity, temperature

## Abstract

Seeds are important materials for the restoration of globally-threatened marine angiosperm (seagrass) populations. In this study, we investigated the differences between different *Ruppia sinensis* seed types and developed two feasible long-term *R. sinensis* seed storage methods. The ability of *R. sinensis* seeds to tolerate the short-term desiccation and extreme cold had been investigated. The tolerance of *R. sinensis* seeds to long-term exposure of high salinity, cold temperature, and desiccation had been considered as potential methods for long-term seed storage. Also, three morphological and nine physiological indices were measured and compared between two types of seeds: Shape L and Shape S. We found that: (1) wet storage at a salinity of 30–40 psu and 0°C were the optimal long-term storage conditions, and the proportion of viable seeds reached over 90% after a storage period of 11 months since the seeds were collected from the reproductive shoots; (2) dry condition was not the optimal choice for long-term storage of *R. sinensis* seeds; however, storing seeds in a dry condition at 5°C and 33 ± 10% relative humidity for 9 months had a relatively high percentage (74.44 ± 2.22%) of viable seeds, consequently desiccation exposure could also be an acceptable seed storage method; (3) *R. sinensis* seeds would lose vigor in the interaction of extreme cold (-27°C) and desiccation; (4) there were significant differences in seed weight, seed curvature, and endocarp thickness between the two types of seeds. These findings provided fundamental physiological information for *R. sinensis* seeds and supported the long-term storage of its seeds. Our results may also serve as useful reference for seed storage of other threatened seagrass species and facilitate their *ex situ* conservation and habitat restoration.

## Introduction

Seagrass meadows are recognized as critical and threatened coastal habitats around the globe. Seagrasses are a unique group of marine angiosperms distributed along the temperate and tropical coastlines of the world (Green and Short, 2003; Short et al., 2007). As important habitat-formers and ecosystem engineers, they form the basis of one of the most widespread and productive coastal ecosystems that provide habitats, foods, and nurseries for a variety of marine organisms (Costanza et al., 1997; Beck et al., 2001; Jackson et al., 2001; Harborne et al., 2006; Heck and Valentine, 2006; Barbier et al., 2011; Liu et al., 2013; Taylor et al., 2017), as well as reducing exposure to bacterial pathogens of humans, fishes, and invertebrates (Lamb et al., 2017). However, seagrass meadows are disappearing at an alarming rate worldwide and facing anthropogenic and natural threats globally (Orth et al., 2006; Waycott et al., 2009; Short et al., 2011, 2016; Maxwell et al., 2017; Unsworth et al., 2017). Thus, effective management and active restoration programs are becoming increasingly important (van Katwijk et al., 2016; Lefcheck et al., 2017).

Numerous studies have considered seagrass restoration and a variety of techniques have been used to restore seagrass beds (Shafer and Bergstrom, 2010; Zhou et al., 2014; van Katwijk et al., 2016). In contrast to the high cost of planting entire shoots and seedlings (Pickerell et al., 2005; Valdemarsen et al., 2010), planting seeds is considered a cost-efficient method for large-scale restoration, and it also maintains high genetic diversity in the restored population (Marion and Orth, 2010; Tanner and Parham, 2010; Reynolds et al., 2013). It is well-known that seed storage can be employed in greenhouse seedling propagation and is a valuable method of *ex situ* conservation.

*Ruppia*, a globally distributed seagrass genus, is also facing large-scale destruction (Copertino et al., 2016). In China, *Ruppia* is mostly found in mariculture and salt ponds (Yu and den Hartog, 2014; Yu et al., 2014). Recent surveys have shown that large areas of *Ruppia* meadows have been destroyed by farmers. Thus, restoration efforts will be important for *Ruppia*. In variable and disturbed environments, the regeneration of the *Ruppia* population is highly dependent on sexual reproduction and seed set. However, although 85% of the seeds germinate, most do not successfully produce seedlings (Strazisar et al., 2016). Thus, it is necessary to build artificial seed banks of *Ruppia* to provide restoration materials.

The ability of seeds to survive desiccation plays an important role in plant regeneration ecology (Tweddle et al., 2003). Seed desiccation tolerance is generally divided into three broad categories: desiccation tolerant (orthodox), intermediate, and desiccation sensitive (recalcitrant). The trait has important implications for species conservation and restoration, as desiccation-sensitive seeds cannot be stored using traditional seed banking techniques. Globally, the majority of flowering plants including Alismatales produce desiccation-tolerant seeds (Wyse and Dickie, 2017). However, the majority of seagrass seeds are suggested to be desiccation sensitive, although little is known about the seed ecology of these species. Pan et al. (2012) suggested that *Zostera marina* seeds are very strongly desiccation sensitive which lose vigor completely after desiccating for 24 h. Cho and Sanders (2009) reported that desiccation greatly reduced the viability of *R. maritima* seeds; the seed viability (35.7%) in dry conditions under ambient temperature (15–25°C) for 10 months was significantly lower than those (>90%) of freshly matured seeds with soft or fully formed seed coats.

Storing seeds in wet conditions has been examined in several seagrass species. Studies of *Zostera* seagrass seed storage suggested that wet conditions at high salinity and cold temperatures can inhibit seed germination (Kaldy et al., 2015; Infantes et al., 2016; Xu et al., 2016). A study of *R. maritima* seed storage showed that both salinity and temperature could be manipulated as storage conditions to retain the vigor of seeds (Ailstock et al., 2010); however, due to germination of seeds during the storage at 4°C and ≤30 psu in their work, the storage condition is not optimal for long-term *Ruppia* seed storage.

*Ruppia* can survive exposure to certain dry conditions but the resulting changes in the internal components have not been reported (Cho and Sanders, 2009; Ailstock et al., 2010). Most plant seeds store energy in the form of carbohydrates, proteins, and lipids (Schwender and Ohlrogge, 2002; Debarre et al., 2006; Kuhn and Grof, 2010). Forms of energy storage in *R. maritima* seeds (fat 2.9% DW and protein 7.8% DW) are similar to the other seagrass seeds, e.g., *Zostera* seeds, which contain lipids <2% dry weight (DW) and starch ∼50% DW (Ballard et al., 2004). Changes in the contents of these ingredients suggest physiological activity in seeds, as it has been proven that protein biosynthesis would decline during seed storage (Cruzgarcia et al., 1995). Moreover, accumulation of carbohydrates and proteins, such as oligosaccharides and heat-shock proteins affects the tolerance of desiccation (Waters et al., 1996; Bailly et al., 2001). Changes in lipid content are associated with the reduction of physiological quality, and reduction in fat content indicates loss of quality during storage (Oliveira et al., 2016).

There are morphological and genetic differences among the *Ruppia* species throughout the world and the new *Ruppia* species widely occurring in northern China is recently identified as *Ruppia sinensis* (Yu and den Hartog, 2014). So far, little is known about the seed ecology of this species. Our recent work indicated that salinity and temperature can significantly affect the germination rates of *R. sinensis* seeds (Gu et al., 2017). The latest national survey in China showed that the population distribution of this species has been decreasing. Thus, it is important to investigate the features of *R. sinensis* seeds and find suitable methods for storing the seeds. According to observations in the wild, there are three different types of *R. sinensis* seeds, which have different sizes and shapes. In this study, we hypothesized the following: (1) different types of *R. sinensis* seeds may have different chemical compositions and different responses to desiccation; and (2) the seeds of *R. sinensis* could adapt to the short-term cooling and desiccation. (3) Low temperature, high salinity, and desiccation may be suitable long-term storage conditions for *R. sinensis* seeds. We obtained fundamental physiological information about *R. sinensis* seeds and their storage, which may facilitate the restoration of *R. sinensis* in its habitat.

## Materials and Methods

### Site Description and Seed Collection

**FIGURE 1:**
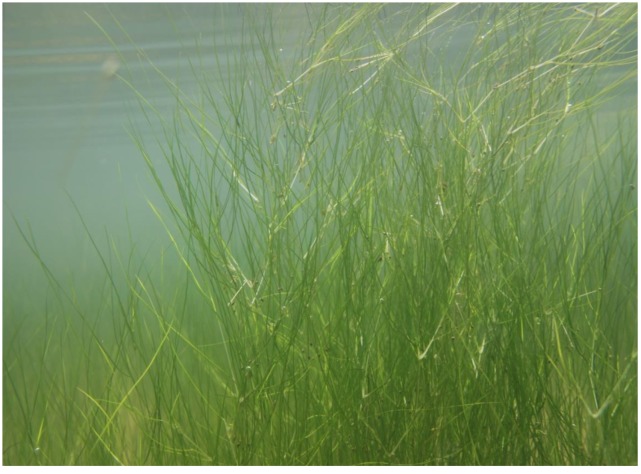
The natural status of *Ruppia sinensis* population in the Dongying area.

The samples were collected in Diaokou Village, Dongying City, Shandong Province (37°59′52″ N, 118°36′33″ W), northern China. The *R. sinensis* population (**Figure 1**) in the site is perennial, and the temperature and salinity range from -4 to 45.8°C and 8 to 32.2 psu, respectively.

*Ruppia sinensis* seeds were collected on September 30, 2016 and they were only taken from reproductive shoots to ensure that all of the seeds were produced in the current year (Kahn and Durako, 2005). All of the samples were brought back to the laboratory on the same day. The shoots were then transferred to large containers of aerated seawater (natural seawater mixed with deionized water with a salinity of 12 psu) and kept at room temperature (around 20°C) in darkness (Koch and Seeliger, 1988) until the exocarps of the seeds decayed naturally and fell to the bottom. After culturing for 28 days, the seeds with black and hard endocarps were selected as mature seeds and temporarily kept in the same seawater conditions aforementioned before experimental use.

### Different Seed Morphology Types

According to field observations, there were three different morphological types of seeds in the population investigated. To determine the proportions of these three types of *R. sinensis* seeds, we randomly collected 16 sediment cores (diameter = 6 cm and depth = 10 cm) during both April and May in 2017. All of the samples were brought back to laboratory and sieved using 0.7-mm meshes. The *R. sinensis* seeds were then selected by hand and we counted the numbers of the different seed types (Riddin and Adams, 2009; Strazisar et al., 2013).

**FIGURE 2:**
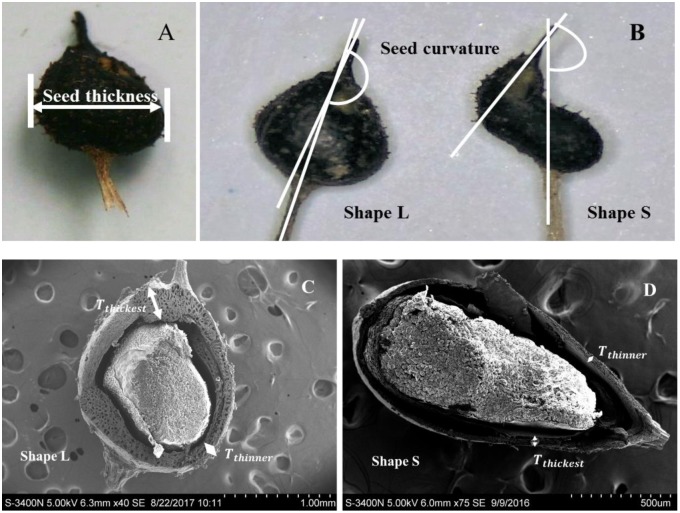
Differences in the morphological measurement indices for Shape L and Shape S *Ruppia sinensis* seeds. **(A)** Seed thickness; **(B)** seed curvature; and **(C,D)** endocarp thickness.

Among the three seed morphology types, the two most common types were the largest and smallest seeds, whereas the medium seed type was the least common. Thus, the first two types of seeds were selected as the experimental materials, which were designated as “Shape L” for the larger seeds and “Shape S” for the smaller seeds. To better distinguish these two types of seeds, 150 seeds of each type were visually selected and we measured the seed thickness and seed curvature. The seeds had the shape of a prolate spheroid and the lengths of the short axes were measured using vernier calipers to determine the seed thickness (**Figure 2A**). The seed curvature was described using the angle created by lines extending from the beak and peduncle (**Figure 2B**). All of the angles were measured with a protractor using seed images captured with a digital camera (precision = 0.01 mm). The thickness of the seed endocarps was measured at the greatest width for 12 seeds (six per seed type) based on an electron microscopic section (**Figures 2C,D**). The thickness of the seed endocarps (*Te*; μm) corresponded to the means of the thickest and the thinnest endocarp areas on one seed:

$$T_{e}=\overset{6}{\underset{i}{\Sigma }}\frac{T_{thickest}+T_{thinnest}}{2}/6$$

where *T_thickest_* represents the thickest measurement of the seed, *T_thinnest_* represents the thinnest measurement of the seed, and *i* represents the seed number.

In addition, the moisture contents of the Shape L and Shape S seeds were measured. Both the wet weight and dry weight (DW) of the seeds were measured, where each of the wet weights was determined based on six replicates (each with 50 seeds). The Shape L and Shape S seeds were dried at 130°C for 1 h and cooled at 30% relative humidity (International Seed Testing Association [ISTA], 1993). The moisture contents (*MC*; %) of the seeds were determined using the following equation:

$$MC=\frac{M_{w}-M_{D}}{M_{w}}\times 100\%$$

where *M_w_* represents the fresh weight in grams of the initial seeds and *M_D_* represents the weight in grams of the dried seeds.

The Shape L and Shape S seeds were also shelled in their initial state, before measuring the carbohydrate, lipid, and protein contents of the embryos. The carbohydrate contents of the seed embryos were determined using a total carbohydrate kit (Beijing Solarbio Science & Technology, Co., Ltd., BC2715), where the carbohydrate was hydrolyzed by H_2_SO_4_, reacted with DNS reagent, and detected as a brownish-red color. The lipids were measured after the samples being freeze-dried. Then the lipids were extracted by chloroform–methanol (1:1) solution at 85°C, and shake-extracted by *n*-hexane at room temperature. The extracting solution was filtered by 0.45 μm filter membrane and injected into a gas chromatographic–mass spectrometer system. The gas chromatograph analytical column was TG-5MS (30 m × 0.25 mm × 0.25 μm) with the injector temperature 290°C and the purge flow 1.20 ml/min; the ionization mode of mass spectrometer was 70 eV EI with the ion source temperature 280°C and the scan range 30–400 amu. The protein contents of the seed embryos were estimated based on the nitrogen contents using the following conversion equation (Delefosse et al., 2016).

[protein]=[N]×6.25

The nitrogen contents were determined by the analyzing seeds after the three treatment conditions (50 seeds per replicate) using a VARIO ELIII elemental analyzer.

To further observe the responses of the internal compositions of both types of *R. sinensis* seeds to short-term desiccation, we selected 150 Shape L seeds and 150 Shape S seeds, and each group was assigned to three replicates (50 seeds per replicate). These seeds were then exposed to desiccating conditions at 40°C and 33 ± 10% relative humidity under continuous darkness, and we measured their seed vigor after 7 days. The seed vigor was tested through the germination experiment. To test germination, each seed treatment was kept in 150 mL glass beakers containing artificial seawater at a salinity of 5 psu in a light incubator at 30°C with a light irradiance at 70 μE m^-2^ s^-1^ and a 12:12 h light: dark photoperiod (Gu et al., 2017). Seeds where the cotyledon emerged were considered germinated seeds (Koch and Seeliger, 1988). Artificial seawater was changed every 4 days.

### Short-Term Desiccation Exposure

**FIGURE 3:**
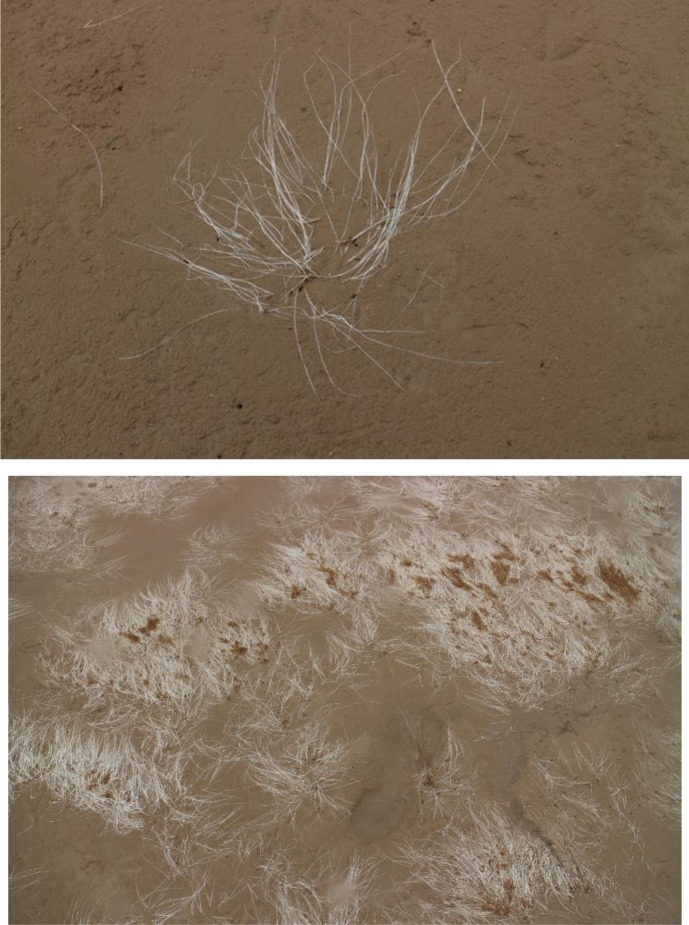
*Ruppia sinensis* containing seeds exposed to desiccation in the Diaokou area.

In the Dongying area, *R. sinensis* is exposed to dry conditions in summer and winter months (**Figure 3**). To replicate these *in situ* conditions and explore the response of *R. sinensis* seeds to dry conditions, we exposed the *R. sinensis* seeds to four different durations (2 h, 8 h, 1 day, and 7 days) and five different temperatures (-27, -10, 0, 26, and 40°C). Before the experiment, initial moisture content (*MC_i_*; %) of the seedlot was determined using the method described above. Each desiccation experimental treatment in 6-cm Petri dishes had three replicates. In total, 30 randomly picked seeds were prepared per replicate and all of the exposure environments were controlled at 33 ± 10% relative humidity under continuous darkness.

Seed vigor was tested in germination tests, which were conducted by monitoring three replicates of 30 seeds per treatment after different dry exposure durations. The control groups contained 90 seeds (30 per replicate) without dry exposure and they were maintained under the germination conditions. The germination conditions comprised placing the seeds in 150 mL glass beakers containing artificial seawater at a salinity of 2 psu and room temperature (26–31°C) with a light irradiance at 70 μE m^-2^ s^-1^ and a 12:12 h light: dark photoperiod. The artificial seawater was changed every 4 days and the numbers of germinated seeds in each beaker were recorded after 2 weeks.

We also measured the wet weights of the *R. sinensis* seeds before and after they were exposed to the different desiccation temperatures (-27, -10, 0, 26, and 40°C) and durations (2 h, 8 h, 1 day, and 7 days). Moisture contents (%) of seeds under these desiccation conditions were then calculated using the following equation:

$$\text{Moisture content}(\%)=\frac{M_{d}-M_{w}\left(1-{MC}_{i}\right)}{M_{d}}\times 100\%$$

where *M_w_* represents the fresh weight in grams of the initial seeds, *MC*_i_ represents initial seed moisture content (%) before desiccation treatment, and *M_d_* represents the fresh weight of the seeds after dry exposure.

### Seed Storage in Wet Conditions

*Ruppia sinensis* seeds were subjected to long-term storage under six different salinity conditions (salinity = 10, 20, 30, 40, 50, and 60 psu) and two different temperatures (0 and 5°C) after storage for 2 months since the seed collection from reproductive shoots. The control group was kept at a salinity of 10 psu and room temperature (10–25°C). Each treatment was maintained in a 1,000-mL glass jar containing ∼1,000 seeds and artificial seawater (sea salt dissolved in deionized water) corresponding to the appropriate salinity level. The bottles were placed in two low-temperature incubators set at 0 and 5°C under continuous darkness. The artificial seawater was changed each month. Germinated and decomposed seeds were regarded as seed losses, which were recorded every 3 months (Strazisar et al., 2013). The percentage seed loss was the proportion of accumulated seed losses relative to the total number of *R. sinensis* seeds in the treatment, as follows:

$$\text{Seed loss}=\frac{n_{i}}{N_{i}}\times 100\%$$

where *n_i_* represents the accumulated number of seeds lost, *N_i_* represents the total number of seeds in the treatment, and *i* represents the treatment number.

To determine the vigor of the seeds, 150 seeds (50 per replicate) were randomly selected from each treatment every 3 months, before testing the seed vigor in the germination test as described above.

Based on the experimental results, a salinity of 30 psu and 0°C were selected as the long-term wet storage conditions. To observe the effects of these wet storage conditions on the compositions of the *R. sinensis* seeds, we determined the moisture content, carbohydrate, lipid, and protein contents of the seeds using the methods described above.

### Seed Storage in Dry Conditions

We performed a long-term seed storage desiccation experiment where the seeds were exposed to three temperature treatments (-1, 5, and 22°C) at 33 ± 10% relative humidity under continuous darkness for 9 months. Each experimental treatment comprised three replicates with 30 randomly picked seeds per replicates and they were placed in 6-cm Petri dishes. The seed vigor was determined after storage for 9 months (11 months since the seed collection from reproductive shoots) based on germination tests. The germination conditions involved that each treatment was kept in 150 mL glass beakers containing artificial seawater at a salinity of 5 psu in a light incubator at 30°C with a light irradiance at 70 μE m^-2^ s^-1^ and a 12:12 h light:dark photoperiod.

The seed vigor test results showed that the *R. sinensis* seeds kept under 5°C storage conditions had relatively high survival percentages (**Figure 9**), thus this temperature range was selected for the long-term desiccation conditions. To observe the effects of long-term dry storage on the internal composition of the *R. sinensis* seeds in this treatment, we measured the moisture content, carbohydrate, lipid, and protein contents of the seeds, as described above.

### Statistical Analyses

Two-way analysis of variance (ANOVA) was employed to compare the effects of the dry exposure durations and dry exposure temperatures in the short-term dry exposure experiment. When the interaction was significant, a simple effect test (a one-way ANOVA and Tukey’s multiple comparisons) was conducted when the effects of dry exposure duration and temperature were both significant (*p* < 0.05) (Zar, 1999). The different morphological indices for the two types of *R. sinensis* seeds, i.e., the seed DWs, embryo DWs, seed thicknesses, seed curvatures, endocarp thicknesses, fatty acid and carbohydrate contents of the dry seeds, and the seed moisture contents after storing in wet or dry conditions, were analyzed using one-way ANOVA. Tukey’s honestly significant difference test was used to identify specific differences between the morphological types and treatments (Riddin and Adams, 2009).

The relationship between the seed weight and endocarp thickness in the two types of *R. sinensis* seeds as well as the relationship between the number of germinated seeds and moisture contents of the seeds after short-term dry exposure were determined by linear regression (*p* < 0.05) using Pearson’s correlation coefficient (r).

We analyzed the indices comprising the proportion in the sediment, moisture content, proportion of endocarp weight in the dry seeds, protein content of the dry seeds, and moisture content after short-term dry exposure after logistic regression of the data and the significant differences were determined using the Cochran–Mantel–Haensel χ^2^ test (Warton and Hui, 2011). Multiple comparisons of the χ^2^ values were used to determine significant differences between the different dry exposure duration and temperature treatments. The seed germination percentages after long-term storage in dry and wet conditions were also tested using the Cochran–Mantel–Haensel χ^2^ test. Differences were considered significant at *p* < 0.05. Statistical analyses were conducted using SAS v.9.2 and SPSS 19.0.

## Results

### Inter-population Seed Differences

**FIGURE 4:**
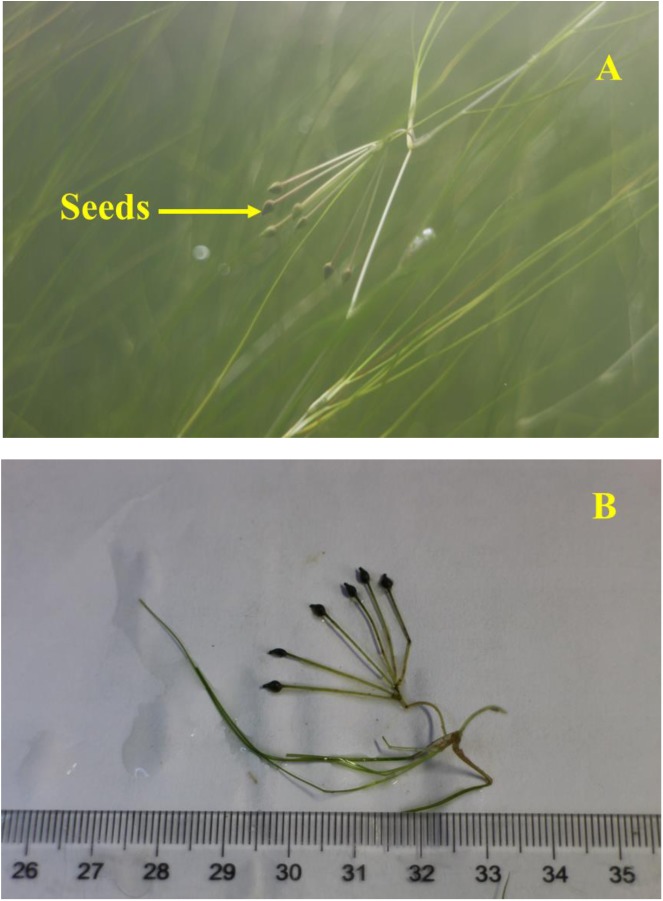
Different types of *Ruppia sinensis* seeds on one rachis. **(A)** Natural state of *R. sinensis* seeds; **(B)** *R. sinensis* seeds measured in the laboratory.

Different types of *R. sinensis* seeds were found in a single rachis (**Figure 4**). The two types of *R. sinensis* seeds accounted for 85.26% of the seeds in the population (**Table 1**; χ^2^ = 1023.411, df = 28, *p* < 0.001). The significant differences in terms of the seed thickness and seed curvature between the two types of seeds indicated that there were obvious morphological differences between the small (Shape S) and large (Shape L) seeds (*p* < 0.001). There were no significant differences in dry embryo weights and changes in their moisture contents between different types of seeds (*p* > 0.05). By contrast, the endocarps were thicker in the Shape L seeds than the Shape S seeds, which contributed to the distinct differences in the DWs of the two seed types (*r* = 0.97, *p* < 0.001, *n* = 11).

**Table 1:** Differences between Shape L and Shape S seeds.

	Proportion in sediment (%)	Seed dry weight (mg)	Dry embryo weight (mg)	Moisture content (%)	Proportion of endocarp weight in dry seed (%)	Seed thickness (mm)	Seed curvature (°)	Endocarp thickness (μm)
Shape L	35.99 ± 5.28^a^	1.50 ± 0.05^a^	0.44 ± 0.03^a^	38.60 ± 2.07^a^	52.09 ± 8.59^a^	1.32 ± 0.02^a^	177.5 ± 1.2^a^	224.50 ± 13.70^a^
Shape S	49.27 ± 4.85^b^	0.67 ± 0.02^b^	0.37 ± 0.02^a^	39.23 ± 0.77^a^	39.24 ± 4.92^b^	0.67 ± 0.01^b^	146.7 ± 1.6^b^	18.75 ± 1.83^b^


*Different letters indicate significant differences at *p* < 0.05 (mean ± SE).*

The carbohydrate and protein contents of the embryos were similar in the two seed types. Also, both the total unsaturated and total saturated fatty acids had no significant statistically differences. Palmitic (C16:0), oleic (C18:1), and linoleic (C18:2) acids were the first three highest content of fatty acids in the dry embryos of *R. sinensis*. However, the contents of palmitic (C16:0), oleic (C18:1), and linoleic (C18:2) acids in the Shape S dry embryo were significant higher than those in the Shape L dry embryo (**Table 2**). Moreover, both types of seeds maintained their seed vigor after 7 days of dry exposure (**Table 2**). Thus, the two types of seeds were significantly different in morphological terms but there were no significant differences in their chemical compositions between them (*p* > 0.05).

**Table 2:** Differences in chemical compositions and germination percentage of the two types of *R. sinensis* seeds.

	Carbohydrate (mg/g)	Protein (%)	Fatty acids (mg/g)					Germination percentage (%)
				
			C16.0	C18.1	C18.2	Total unsaturated	Total saturated	
Shape L	478.34 ± 68.06^a^	16.95 ± 0.76^a^	11.66 ± 0.46^b^	76.89 ± 2.61^b^	134.25 ± 7.32^b^	211.40 ± 9.95^a^	17.92 ± 0.76^a^	53.33 ± 3.84^a^
Shape S	543.48 ± 56.39^a^	17.89 ± 0.48^a^	17.38 ± 1.83^a^	104.26 ± 12.26^a^	206.25 ± 23.78^a^	310.66 ± 36.09^a^	25.77 ± 2.75^a^	53.33 ± 11.76^a^


*Different letters indicate significant differences at *p* < 0.05 (mean ± SE). Carbohydrate represents the carbohydrate concentration in the dry embryo. Protein represents the proportion of protein relative to the embryo’s dry weight. Fatty acids represent the fatty acid concentrations in the dry embryo. Germination percentage (%) was determined after desiccation exposure for 7 days.*

### Short-Term Dry Exposure

**FIGURE 5:**
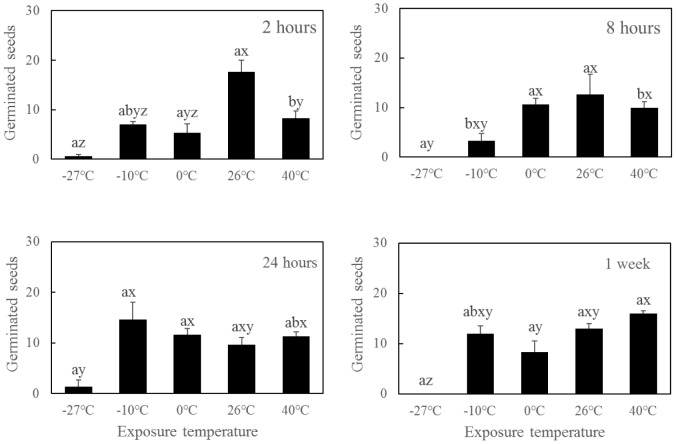
Germination of *Ruppia sinensis* seeds after short-term dry exposure at a salinity of 2 psu and room temperature (26–31°C) conditions (mean ± SE). Different letters a and b indicate significant differences among different dry exposure durations at each temperature. Different letters x, y, and z indicate significant differences among different temperatures at each exposure duration. The data were analyzed using one-way ANOVA and Tukey’s multiple comparisons test (*p* < 0.05).

Both the dry exposure duration and temperature had significant effects on seed survival and germination (**Table 3**). Short-term dry exposure at temperatures above -10°C maintained the seed vigor, whereas the seeds treated with extreme cold conditions (-27°C) exhibited decreased seed viability after dry exposure for 2 h (**Figure 5**). Desiccation in extreme cold conditions also decreased the survivability of *R. sinensis* seeds.

**Table 3:** Statistical differences in the effects of exposure temperature and duration on the number of germinated seeds.

Variable	df	Sum square	Mean square	*F*-value	*p* (>F)
**Two-way ANOVA**					
Model	20	1765.524	88.276	10.493	<0.001
Exposure temperature	4	1148.567	287.142	34.132	<0.001
Exposure duration	3	76.583	25.528	3.034	0.047
Exposure temperature × exposure duration	12	425.167	35.431	4.212	<0.001
**Simple effects**					
Different exposure durations at each exposure temperature (°C)					
-27	3	3.667	1.222	0.863	0.499
-10	3	230.917	76.972	6.326	0.017
0	3	71.333	23.778	2.853	0.105
26	3	98.250	32.750	1.747	0.235
40	3	97.583	32.528	9.520	0.005
Different exposure temperature at each exposure duration					
2 h	4	456.733	116.433	16.956	<0.001
8 h	4	349.333	87.333	6.823	0.006
24 h	4	303.600	75.900	7.206	0.005
7 days	4	455.067	113.767	22.454	<0.001


*One-way ANOVA and Tukey’s multiple comparisons were used to test the simple effects of dry exposure duration at each temperature, and the simple effects of different temperature at each dry exposure duration.*

**FIGURE 6:**
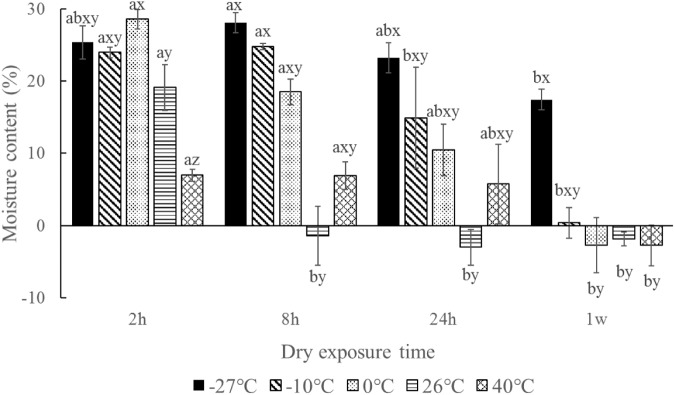
Moisture content of *Ruppia sinensis* seeds after dry exposure at different temperatures and durations. The initial seed moisture content was 37.76 ± 1.80%. Different letters a and b indicate significant differences among different dry exposure durations at each temperature. Different letters x, y, and z indicate significant differences among different dry exposure temperatures at each exposure duration. The data were analyzed based on χ^2^ multiple comparisons (*p* < 0.05).

The moisture contents of the seeds after desiccation for 7 days among different temperatures were significant different (*p* < 0.001). An extreme cold temperature could maintain the seed moisture level (**Figure 6**). In contrast, seed moisture contents were almost 0 at higher temperatures (26 and 40°C). The number of germinated seeds was significantly negatively correlated with moisture content (*r* = 0.533, *p* < 0.001, *n* = 60).

### Long-Term Storage in Wet Conditions

**FIGURE 7:**
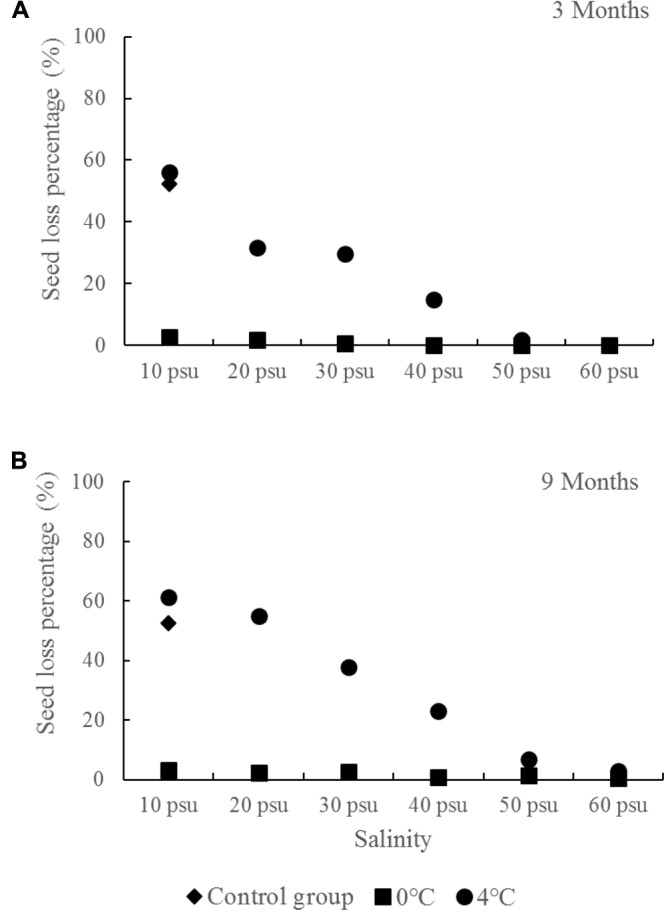
*Ruppia sinensis* seed losses under different salinity and temperature conditions in the long-term wet storage experiments (**A**, 3 months; **B**, 9 months). The control group was the seeds kept at a salinity of 10 psu and room temperature (10–25°C).

The control *R. sinensis* seeds germinated rapidly at a salinity of 10 psu and under variable room temperature (10–25°C) conditions, where the percentage seed loss was 52.21%, which indicated that about half did not undergo dormancy (**Figure 7**). This also suggests that half the seeds would be lost without artificial storage measures. Temperatures of 0 and 4°C are too low for seed germination, but there were significant differences at these temperatures when used for long-term storage. There was a high seed loss percentage at 4°C with a salinity of less than 50 psu, but the highest seed loss occurred at 4°C with a salinity of 10 psu, with 56.06% after 3 months and 61.08% after 9 months. By contrast, all of the salinity treatments at 0°C obtained lower seed loss percentages. Compared with the seeds stored at 4°C, the seeds stored at 0°C were also less likely to be infected by bacteria.

**FIGURE 8:**
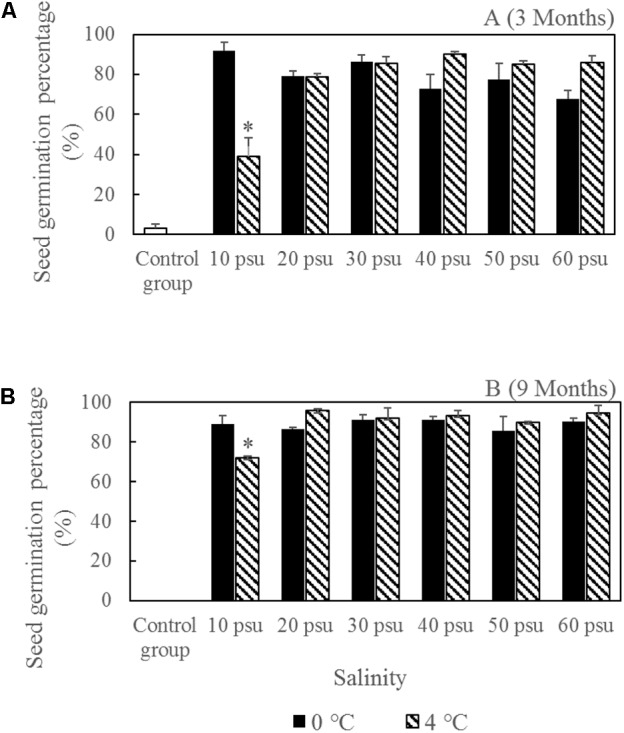
*Ruppia sinensis* seed vigor after storage for 3 months **(A)** and 9 months **(B)** under different salinities and temperatures. ^∗^Significant difference under the same salinity treatment at two different temperatures. The control group was kept at a salinity of 10 psu and room temperature (10–25°C).

**FIGURE 9:**
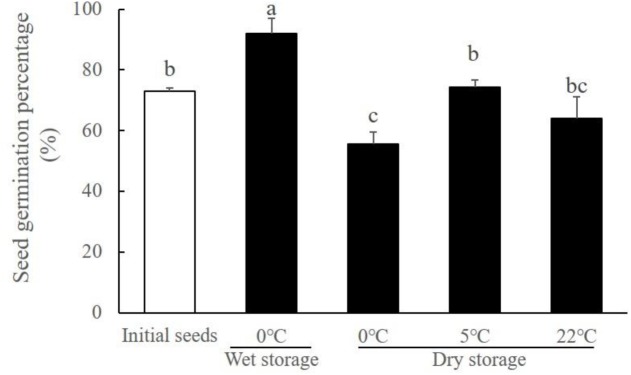
*Ruppia sinensis* seed vigor after storage for 9 months (Black bars) in dry conditions at different temperatures and in wet conditions at 0°C and 30 psu. The white bar represents initial seed germination percentage before the storage (Gu et al., 2017). Different letters a, b, and c indicate significant difference between treatments. All seed germination percentage was determined under the optimal germination conditions (30°C and 5 psu).

The seed vigor was tested in seed germination experiments (**Figure 8**). All of the storage treatments had greater seed vigor levels than the control groups (seed storage at salinity 10 psu and room temperature). The highest seed germination percentage after wet storage for 9 months was 95.70 ± 1.17%, which was obviously higher than the initial germination percentage (73.08 ± 0.90%) under the same germination conditions without storage (Gu et al., 2017) (**Figure 9**). Moreover, the seed vigor levels in most of storage treatments were similar at 0 and 4°C. However, seed storage at 4°C had a significantly higher seed loss compared with storage at 0°C when salinity ≤40 psu (**Figure 7**). Considering the seed loss percentage during storage, seed vigor after storage, and storage conditions, we found that a salinity of 30–40 psu and 0°C were the optimal conditions for the wet storage of *R. sinensis* seeds. After storage at salinity 30–40 psu for 9 months, the seed moisture content decreased from initial 37.76 ± 1.80% to 28.27 ± 0.28% (**Table 4**). The carbohydrate, the total unsaturated fatty acids, the total saturated fatty acids and protein in the dry embryos after stored for 9 months in the optimal wet-condition were 507.94 ± 20.55 mg/g, 97.37 ± 3.13 mg/g, 6.00 ± 0.43 mg/g, and 10.16 ± 0.37%, respectively (**Table 4**).

**Table 4:** Differences in the chemical compositions after storage of *R. sinensis* seeds in wet and dry conditions.

	Moisture content (%)	Carbohydrate (mg/g)	Protein (%)	Fatty acids (mg/g)				
				
				C16.0	C18.1	C18.2	Total unsaturated	Total saturated
Wet-storage seeds	28.27 ± 0.28^a^	507.94 ± 20.55^a^	10.16 ± 0.37^a^	3.88 ± 0.35^a^	26.96 ± 0.67^a^	70.28 ± 2.47^a^	97.37 ± 3.13^a^	6.00 ± 0.43^a^
Dry-storage seeds	11.57 ± 1.48^b^	533.97 ± 10.18^a^	13.11 ± 1.94^a^	2.80 ± 0.08^b^	22.73 ± 0.89^b^	66.73 ± 3.80^a^	89.62 ± 4.70^a^	4.67 ± 0.17^b^


*Different letters indicate significant differences at *p* < 0.05 (mean ± SE). Carbohydrate represents the carbohydrate concentration in the dry embryos. Protein represents the proportion of protein relative to the embryos’ dry weight. Fatty acids represent the fatty acid concentrations in the dry embryos.*

### Long-Term Storage in Dry Conditions

Temperature affected the seed vigor during the dry storage of *R. sinensis* seeds (χ^2^ = 12.094, df = 11, *p* = 0.002). Compared with the optimal long-term storage condition (Salinity 30, 0°C), desiccation exposure reduced the proportion of viable seeds during the seed storage period. Nevertheless, *R. sinensis* seeds stored under the dry conditions at both temperature 5 and 22°C still had a considerable proportion of viable seeds, i.e., 74.44 ± 2.22% and 63.91 ± 7.21%, respectively (**Figure 9**).

Furthermore, there were no significant differences in the chemical compositions of the seeds stored under the optimal dry and wet conditions after storage for 9 months (**Table 4**). The contents of linoleic (C18:2) acids and total unsaturated fatty acids in the seeds stored in the two conditions were also similar, however, palmitic (C16:0), oleic (18:1) acids, and total saturated fatty acids contents of those seeds were significant different (**Table 4**).

## Discussion

In this study, we tested the adaptability of *R. sinensis* seeds to desiccation and different temperatures. We found that *R. sinensis* seeds could tolerate low temperature and high salinity conditions, indicating a potential method for long-term seed storage. Although long-term desiccation could reduce the vigor of *R. sinensis* seeds, it could also be an acceptable seed storage method for ca. 1 year. In addition, we firstly reported the occurrence of two different seed types in the seagrass *R. sinensis* from both morphology and physiology.

The usual low temperature range for storing seagrass seeds in wet conditions is 4–7°C (Ambo-Rappe and Yasir, 2015; Kaldy et al., 2015; Kauth and Biber, 2015; Xu et al., 2016). However, we observed that storing *R. sinensis* seeds at 4°C could delay seed germination for only 1 month, thus it is a suitable temperature for short-term storage of *R. sinensis* seeds (Gu et al., 2017). Furthermore, we found that *R. sinensis* seeds had the ability to tolerate sub-zero temperatures (-10°C), and the germination percentage of seeds stored in such condition for a short term had no significant difference with that exposed in temperature over 0°C (**Figure 5**).

Around 70% of *R. sinensis* seeds can germinate after their exocarps break (Gu et al., 2017). However, the germination percentages were greater for seeds stored under wet conditions for 3 and 9 months (**Figure 8**). This indicates that a small proportion of the *R. sinensis* seeds underwent a period of physiological dormancy and this dormancy could be broken by a low temperature, which was similar to *R. maritima* and *Vallisneria americana* seeds (Ailstock et al., 2010; Kauth and Biber, 2015). Exposure of *R. maritima* seeds in dry conditions under ambient temperature (15–25°C) for 10 months reduces the seed germination proportion from >90 to 35.7% (Cho and Sanders, 2009); in contrast, a relatively high percentage (74.44 ± 2.22%) of the *R. sinensis* seeds survived storage under dry conditions at 5°C for 9 months. The germination percentage of these seeds was somewhat lower than that of the seeds stored under wet conditions, but dry storage is more convenient. Previous studies have suggested that the carbohydrate and protein contents of the seeds may indicate the level of seed vigor (Angelovici et al., 2010; Pammenter and Berjak, 2014). The biosynthesis of proteins decreases during seed storage where the membranes are altered (Bray and Chow, 1976; Finch-Savage and Leubner-Metzger, 2006). Changes in the oligosaccharide content are also related to seed vigor (Bailly et al., 2001). The carbohydrate and protein contents of the *R. sinensis* seeds were measured after storage under different conditions for 9 months. The similarity in fatty acid, protein, and carbohydrate contents (**Table 4**) indicated similar seed vigor after storage. Although, the contents of palmitic (C16:0), oleic (C18:1) acids and the total unsaturated fatty acids in the wet-storage seeds were higher than in the dry-storage seeds, the contents of linoleic (C18:2) acids and the total saturated fatty acids in dry-storage seeds had no statistics differences. Thus, it is feasible to store *R. sinensis* seeds at 0°C and a salinity of 30–40 psu, and to desiccate them at 5°C with 33 ± 10% relative humidity.

Like other angiosperm plants, most seagrasses produce seeds that vary in size among different taxa (Zipperle et al., 2009; Van Tussenbroek et al., 2016; Zhang et al., 2016). The seeds of *Z. marina* differ in size among different geographical areas or local sampling sites (Wyllie-Echeverria et al., 2003; Delefosse et al., 2016). However, seeds with different sizes and shapes are uncommon in a single seagrass population. We found that the population of *Ruppia* in Dongying, northern China had three different types of seeds, with two main types (**Table 1**). The endocarp thickness and seed curvature were the most significant morphological differences between the two seed types. The seed types had similar embryo DWs and moisture contents, thus the difference in seed thickness may be due to variations in the endocarp thickness. Unlike the differences in the chemical compositions between two seed sizes found in another species (Delefosse et al., 2016), we found that the carbohydrate and protein contents were similar in the embryos of the dry seeds (**Table 2**). Both the total unsaturated and total saturated fatty acids were similar in the two types of *R. sinensis* seeds, and the contents of total unsaturated fatty acids were much more than the total saturated (**Table 2**). These results made a further supplement to the conclusion that the presences of unsaturated fatty acids had been correlated with seed desiccation tolerance, and higher proportions of unsaturated fatty acids were founded in the desiccation-sensitive seeds (Liu et al., 2006). There were no significant differences between the two seed types in terms of their tolerance of desiccation (**Table 2**). However, some related components of lipids, carbohydrates, and proteins, including raffinose and antioxidant enzymes, require further study (Bailly et al., 2001; Wang et al., 2017). The differences between the responses of the two seed types to environmental conditions such as extreme cold and long-term desiccation also need to be investigated. The occurrence of two seed types of *R. sinensis* might indicate a reproduction and maintenance strategy of the population, such as producing more small seeds (Shape S) in poor nutritional year and more large seeds (Shape L) in the nutritional adequacy year; otherwise small seeds may have advantages for disperse and transportation.

## Conclusion

Two types of *R. sinensis* seeds were defined using quantitative indicators, and we measured the carbohydrate, fatty acid and protein contents of the two different types of seeds. However, excluding the endocarp thickness, there were no significant differences between the two seed types. *R. sinensis* seeds showed relatively great adaptability to desiccation; however, some seeds lost vigor in the interaction of both desiccation and extreme cold temperature (-27°C). Eventually, we developed two potential methods for the long-term storage of *R. sinensis* seeds. These results provide insights into *R. sinensis* seeds but further research on the mechanisms of the occurrence of two different types of seeds and their physiological differences is required.

## Author Contributions

RG conceived and designed the laboratory experiments, performed the experiments, analyzed and interpreted the data, contributed reagents, materials and analysis tools, wrote the paper, prepared the figures and tables, reviewed drafts of the paper and approved of the submitted and final versions. YZ conceived and designed all the experiments, interpreted the data, revised and reviewed drafts of the paper, and approved of the submitted and final versions. XS, ScX, XZ, HL, SX, SY, and SZ acquired the data, revised the paper, and approved of the submitted and final versions.

## Conflict of Interest Statement

The authors declare that the research was conducted in the absence of any commercial or financial relationships that could be construed as a potential conflict of interest.
